# Robustly printable freeform thermal metamaterials

**DOI:** 10.1038/s41467-021-27543-7

**Published:** 2021-12-10

**Authors:** Wei Sha, Mi Xiao, Jinhao Zhang, Xuecheng Ren, Zhan Zhu, Yan Zhang, Guoqiang Xu, Huagen Li, Xiliang Liu, Xia Chen, Liang Gao, Cheng-Wei Qiu, Run Hu

**Affiliations:** 1 State Key Laboratory of Digital Manufacturing Equipment and Technology, Huazhong University of Science and Technology, Wuhan, 430074 China; 2 State Key Laboratory of Coal Combustion, School of Energy and Power Engineering, Huazhong University of Science and Technology, Wuhan, 430074 China; 3 Department of Electrical and Computer Engineering, National University of Singapore, Kent Ridge, 117583 Republic of Singapore; 4 School of Electrical and Electronic Engineering, Huazhong University of Science and Technology, Wuhan, 430074 China

**Keywords:** Metamaterials, Materials for devices, Structural materials, Thermodynamics

## Abstract

Thermal metamaterials have exhibited great potential on manipulating, controlling and processing the flow of heat, and enabled many promising thermal metadevices, including thermal concentrator, rotator, cloak, etc. However, three long-standing challenges remain formidable, i.e., transformation optics-induced anisotropic material parameters, the limited shape adaptability of experimental thermal metadevices, and a priori knowledge of background temperatures and thermal functionalities. Here, we present robustly printable freeform thermal metamaterials to address these long-standing difficulties. This recipe, taking the local thermal conductivity tensors as the input, resorts to topology optimization for the freeform designs of topological functional cells (TFCs), and then directly assembles and prints them. Three freeform thermal metadevices (concentrator, rotator, and cloak) are specifically designed and 3D-printed, and their omnidirectional concentrating, rotating, and cloaking functionalities are demonstrated both numerically and experimentally. Our study paves a powerful and flexible design paradigm toward advanced thermal metamaterials with complex shapes, omnidirectional functionality, background temperature independence, and fast-prototyping capability.

## Abstract (web summary)

Thermal metamaterials can be used to manipulate heat flow but experimental fabrication is challenging. Here, the authors report robustly printable freeform thermal metamaterials to tackle this challenge by topology optimization and 3D printing.

## Introduction

Metamaterials, due to their extraordinary properties, high design degrees of freedom, abundant physical intensions, and emerging functionalities, have penetrated into almost all disciplines and evolutionally transformed the way we design artificially structured materials, manipulate physical fields, and explore the unknown boundaries. With the prevailing design paradigms like transformation optics^1,2^ and scattering cancellation^3,4^ methods, many novel metamaterials have been proposed in various physical fields, e.g., electromagnetics^5^, acoustics^6^, dc field^7^, elastic mechanics^8^, and thermotics^9–13^. As a diffusive counterpart, thermal metamaterials have exhibited great potentials on manipulating, controlling, and processing the heat flow, and enabled many promising thermal metadevices, including thermal cloak^14–17^, concentrator^16,18^, rotator^18,19^, camouflage^20–22^, and illusion^20,23^, etc. However, three long-standing challenges remain formidable for state-of-the-art thermal metamaterials^13,24–27^. Firstly, the transformation optics method results in thermal metamaterials with inhomogeneous and anisotropic material parameters that are hard to realize by naturally existing materials. Secondly, thermal metadevices, especially thermal cloak, thermal concentrator, thermal rotator, are usually fabricated in experiments by layered strategies with concentric circular^18^ or elliptical ring-like structures^28^, which may limit the shape adaptability toward practical application. Thirdly, most direct optimization methods^29–33^ employed in the design of thermal metamaterials need to involve and evaluate the preset background temperature (BT) fields or thermal functionalities in the optimization process, which in turn affects the design efficiency, flexibility, and versatility.

**Fig. 1:**
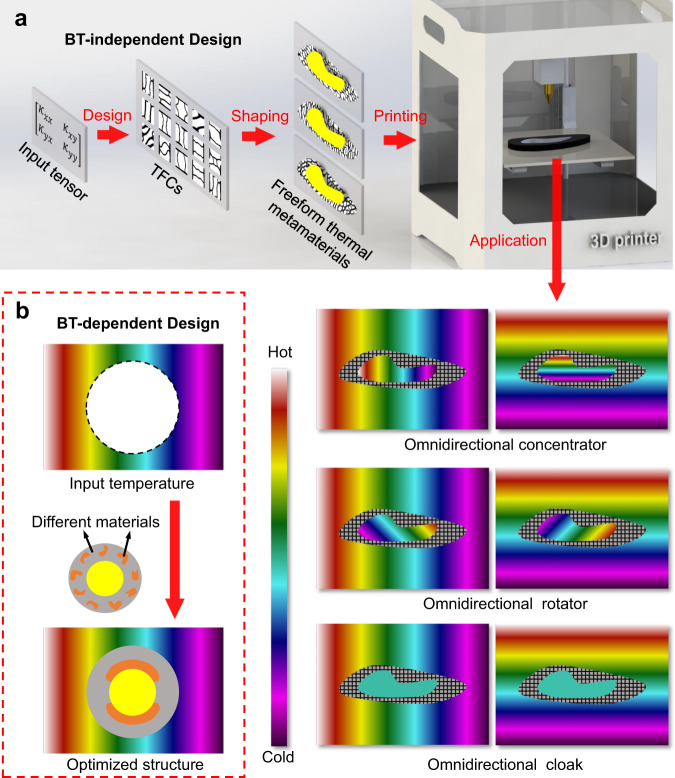
Schematic of BT-independent and BT-dependent design paradigms. **a** BT-independent design paradigm is started by inputting the thermal conductivity tensor into structural topology optimization for TFCs, which are then shaped into freeform thermal metamaterials and fabricated by 3D printing. The omnidirectional functionalities are demonstrated by the three ideal temperature fields with embedded thermal concentrator, rotator and cloak, when heat flow is launched from arbitrary directions. **b** BT-dependent design paradigm takes background temperatures as input reference, and the distribution of different materials is optimized to make the resulting temperature field close to the reference one. The yellow region denotes an object.

According to the general Fermat’s principle, heat flow follows the path of the least thermal resistance, i.e., heat flow tends to flow along the materials with high thermal conductivity^34^. Therefore, one can design local materials to guide the heat flow and then assemble them accordingly to achieve desired temperature profiles and thermal functionalities. The requirement of the local materials, typically the thermal conductivity distribution, can be quantified by theoretical predictions like transformation optics and scattering cancellation methods, and the remaining task is to find or fabricate the right local materials with the corresponding local properties. When the local materials with the desired thermal properties cannot be found in nature, mixing of different materials may be a solution, but how to design the corresponding volume fraction and material distribution is challenging, let alone the nearly inevitable thermal contact resistance problem. A feasible way to tackle such challenges is to employ numerical optimization algorithms^29–33,35,36^, like topology optimization^29–33^. However, most topology optimization strategies are used to design the thermal metamaterials by optimizing the global material distribution. As shown in Fig. 1(b), by taking the preset temperature distribution as input reference, the distribution of different materials in the region of interest is optimized to converge the temperature field toward the reference one under the same boundary conditions. Note that such numerical methods inevitably require a priori knowledge of the BT, and are thus called as BT-dependent design methods hereinafter. Such BT-dependent design methods may not be applicable when the BTs cannot be measured or keep changing.

In this work, we propose a BT-independent design paradigm for robustly printable freeform thermal metamaterials, which tackles the above three challenges at one go. As shown in Fig. 1(a), we firstly calculate the desired thermal conductivity tensor by transformation optics method, and then discretize the whole domain into individual topological functional cells (TFCs) with local microstructures, which are determined by the topology optimization method using two constituent materials—Die steel (H13) and polydimethylsiloxane (PDMS). Since the local thermal conductivities vary from TFC to TFC, the microstructures are also distinct from each other. To showcase the powerfulness of our approach, we then arrange and fabricate the TFCs into freeform thermal concentrator, rotator and cloak via 3D printing technique, which has been adopted to manufacture the recent freeform metasurfaces^37,38^ and metamaterials^39^. The ideal steady-state temperature field of three freeform thermal metadevices under omnidirectional heat input are also illustrated as typical examples in Fig. 1(a), respectively. The influence of the object to the external temperature profiles will be removed no matter from which direction the heat flows across the object, while the temperature gradient inside the object can be cloaked, concentrated, and rotated flexibly depending on the desired functionalities. This study enables the powerful and flexible BT-independent design paradigm of thermal metamaterials, and triggers more explorations on other thermal functionalities and physical fields.

## Results

### Design of the robustly printable freeform thermal metamaterial

BT-independent design for printable freeform thermal metamaterials includes four steps. Firstly, according to the desired functionalities and the shape of the thermal metadevices, we calculate the required thermal conductivity tensor distribution in the metadevice region. Secondly, we grid the metadevice region into small discrete TFCs (as small as possible for higher precision) that hold the calculated thermal conductivity tensors accordingly. Thirdly, we take the required thermal conductivity tensor as the goal and design the topological configuration of each TFC by topology optimization. Finally, we assemble all the TFCs in a freeform fashion to realize the targeted thermal functionalities.

**Fig. 2:**
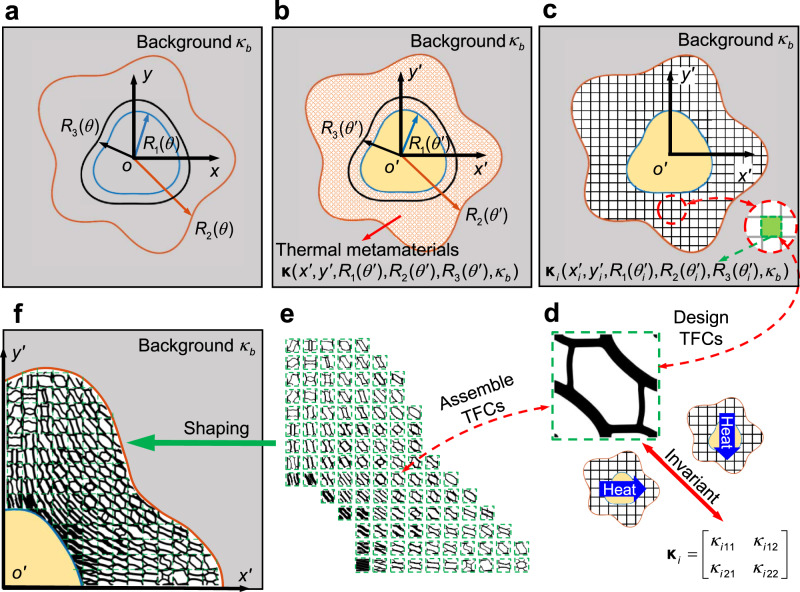
Stepwise roadmap of BT-independent design paradigm for the robustly printable freeform thermal metamaterials. **a** A pure background with a thermal conductivity ${\kappa }_{{{{{{\rm{b}}}}}}}$. Three arbitrary-shape curves ${R}_{1}(\theta )$, ${R}_{2}(\theta )$, ${R}_{3}(\theta )$ in cylindrical coordinate system denote the design regions in the virtual space. **b** Corresponding regions in the real space to denote the object and the metadevice by geometric mapping between (**a**) and (**b**). The yellow region denotes an object. The region shaded with red lines between ${R}_{1}(\theta {\prime} )$ and ${R}_{2}(\theta {\prime} )$ is filled with thermal metamaterials and the local thermal conductivity tensor is dependent on the position $(x{\prime} ,y{\prime} )$, the shape ${R}_{1}(\theta {\prime} )$, ${R}_{2}(\theta {\prime} )$, ${R}_{3}(\theta {\prime} )$, and background conductivity ${\kappa }_{{{{{{\rm{b}}}}}}}$. **c** Schematic for designing the TFCs. The metamaterial region is divided into many small square TFCs whose thermal conductivity tensor ${\kappa }_{i}({x{\prime} }_{i},{y{\prime} }_{i},{R}_{1}({\theta {\prime} }_{i}),{R}_{2}({\theta {\prime} }_{i}),{R}_{3}({\theta {\prime} }_{i}),{\kappa }_{{{{{{\mathrm{b}}}}}}})$ is calculated from the central point of each TFC and is the design goal for the subsequent structural topology optimization. **d** The invariant thermal conductivity tensor when the heat flows across the TFCs in different directions. **e** Schematic of the TFCs in a quarter region. **f** Schematic of thermal metadevice shaped by assembling the TFCs.

We start with the calculation of the thermal conductivity tensors for each point in the metadevice region via the transformation optics method. The Laplacian heat conduction equation in the virtual/original space without heat sources at the steady state is $\nabla \cdot ({{{{{{\boldsymbol{\kappa }}}}}}}_{v}\nabla T)=0$, where ${{{{{{\boldsymbol{\kappa }}}}}}}_{v}$ is thermal conductivity tensor and *T* is temperature. By transforming the cylindrical coordinate in the original/virtual space $(r,\theta )$ into the transformed/real space $(r^{\prime} ,\theta^ {\prime} )$, the governing equation of thermal conduction in the transformed/real space maintains its form as $\nabla^{\prime} \cdot ({{{{{{\boldsymbol{\kappa }}}}}}^{{{\prime} }}}_{R}\nabla^{\prime} T^{\prime} )=0$. According to the transformation optics theory, the transformed thermal conductivity tensor ${{{{{{\boldsymbol{\kappa}}}}}}^{\prime }}_{R}$ in real space can be calculated as ${{{{{{\boldsymbol{\kappa }}}}}}^{{{\prime} }}}_{R}={{{{{\bf{J}}}}}}^{{{\prime} }}{\kappa }_{{{{{{\mathrm{b}}}}}}}{{{{{{\bf{J}}}}}}^{{{\prime} }}}^{T}/\det {{{{{\bf{J}}}}}}^{{{\prime} }}$, where ${{{{{\bf{J}}}}}}^{{{\prime} }}=\partial (r^{\prime} ,\theta^{\prime} )/\partial (r,\theta )$ is the Jacobian matrix of the coordinate transformation and ${\kappa }_{{{{{{\mathrm{b}}}}}}}$ is the homogeneous thermal conductivity of background. In Fig. 2(a, b), we map the arbitrary-shape metadevice in the real space into a homogeneous plate in the virtual space, and the transformed thermal conductivity tensors of the metadevices, including thermal concentrator, rotator and cloak, can be obtained in Cartesian coordinate system, respectively, as $${{{{{{\boldsymbol{\kappa }}}}}}}^{A}=\left[\begin{array}{cc}{\kappa }_{11}^{A} & {\kappa }_{12}^{A}\\ {\kappa }_{21}^{A} & {\kappa }_{22}^{A}\end{array}\right]$$, $${{{{{{\boldsymbol{\kappa }}}}}}}^{B}=\left[\begin{array}{cc}{\kappa }_{11}^{B} & {\kappa }_{12}^{B}\\ {\kappa }_{21}^{B} & {\kappa }_{22}^{C}\end{array}\right]$$, and $${{{{{{\boldsymbol{\kappa }}}}}}}^{C}=\left[\begin{array}{cc}{\kappa }_{11}^{C} & {\kappa }_{12}^{C}\\ {\kappa }_{21}^{C} & {\kappa }_{22}^{C}\end{array}\right]$$, which are dependent on the position $(x{\prime} ,y{\prime} )$, the shape curves ${R}_{1}(\theta ^{\prime} )$, ${R}_{2}(\theta ^{\prime} )$, and ${R}_{3}(\theta ^{\prime} )$, and the background conductivity ${\kappa }_{{{{{{\mathrm{b}}}}}}}$. Details of the deduction processes for the required thermal conductivity tensors of the three metadevices can be found in Supplementary Note 1.

Note that these thermal conductivity tensors of the arbitrary-shape metadevices between ${R}_{1}(\theta ^{\prime} )$ and ${R}_{2}(\theta ^{\prime} )$ are strongly anisotropic and inhomogeneous, which are so anisotropic that it is rather challenging to be achieved by the alternative layers of thermal insulator and conductor strategy experimentally^18,21,28^. An alternative method to achieve such anisotropic thermal conductivity tensor is to optimize the corresponding volume fractions and material distributions of different materials by topology optimization method^40^. In this regard, as shown in Fig. 2(c), we firstly divide the metadevice region into many small TFCs, whose thermal conductivity tensor $${\kappa }_{ilm}^{{{{{{\rm{Input}}}}}}}=\left[\begin{array}{cc}{\kappa }_{i11}^{{{{{{\rm{Input}}}}}}} & {\kappa }_{i12}^{{{{{{\rm{Input}}}}}}}\\ {\kappa }_{i21}^{{{{{{\rm{Input}}}}}}} & {\kappa }_{i22}^{{{{{{\rm{Input}}}}}}}\end{array}\right](l,m=1,2)$$ is calculated by substituting the central point of the TFCs into ${{{{{{\boldsymbol{\kappa }}}}}}}_{i}({x{\prime} }_{i},{y{\prime} }_{i},{R}_{1}({\theta ^{\prime} }_{i}),{R}_{2}({\theta ^{\prime} }_{i}),{R}_{3}({\theta ^{\prime} }_{i}),{\kappa }_{{{{{{\mathrm{b}}}}}}})$ and taken as the input for the subsequent topology optimization. Then, we assume periodic boundary conditions for each TFC and mesh each TFC into *N* finite elements to calculate its equivalent macroscopic thermal property. The homogenized thermal conductivity tensor of *i*th TFC $${\kappa }_{ilm}^{{{{{{\rm{Output}}}}}}}=\left[\begin{array}{cc}{\kappa }_{i11}^{{{{{{\rm{Output}}}}}}} & {\kappa }_{i12}^{{{{{{\rm{Output}}}}}}}\\ {\kappa }_{i21}^{{{{{{\rm{Output}}}}}}} & {\kappa }_{i22}^{{{{{{\rm{Output}}}}}}}\end{array}\right](l,m=1,2)\,$$can be output under the framework of the finite element method (FEM) as^41,42^

1 $${\kappa }_{ilm}^{{{{{{\rm{Output}}}}}}}=\frac{1}{|V|}\mathop{\sum }\limits_{e=1}^{N}{(\varDelta {{{{{{\bf{T}}}}}}}_{e}^{(l)})}^{T}{{{{{{\bf{k}}}}}}}_{e}({\rho }_{e})\varDelta {{{{{{\bf{T}}}}}}}_{e}^{(m)}$$

where $|V|$ is the total volume of *i*th TFC. $\varDelta {{{{{{\bf{T}}}}}}}_{e}(={{{{{{\bf{T}}}}}}}_{e}^{0}-{{{{{{\bf{T}}}}}}}_{e})$ is the temperature vector difference where ${{{{{{\bf{T}}}}}}}_{e}^{0}$ is the nodal temperature vector under the uniform test heat flow ${{{{{{\bf{q}}}}}}}_{e}^{0}$ (e.g. ${\{1,{{{{{\rm{0}}}}}}\}}^{T}$ and ${\{0,{{{{{\rm{1}}}}}}\}}^{T}$ in 2D case), and **T**_*e*_ is the induced nodal temperature field resulting from finite element analysis (FEA) of the base mesh element. ${{{{{{\bf{k}}}}}}}_{e}({\rho }_{e})=\kappa ({\rho }_{e}){{{{{{\bf{k}}}}}}}_{e}^{0}$ is the thermal conductivity matrix determined by the thermal conductivity coefficient $\kappa ({\rho }_{e})$ of each finite element and the unit thermal conductivity matrix ${{{{{{\bf{k}}}}}}}_{e}^{0}={\int }_{{V}_{e}}[{(\frac{\partial {{{{{\bf{N}}}}}}}{\partial x})}^{T}(\frac{\partial {{{{{\bf{N}}}}}}}{\partial x})+{(\frac{\partial {{{{{\bf{N}}}}}}}{\partial y})}^{T}(\frac{\partial {{{{{\bf{N}}}}}}}{\partial y})]{{{{{\mathrm{d}}}}}}{V}_{e}$, where **N** is the shape function in FEA and *V*_e_ is the volume of a finite element. Each finite element is assigned an artificial continuous design variable ${\rho }_{e}$, which is defined in a range from material 1 (${\rho }_{e}=0$) to material 2 (${\rho }_{e}=1$). Here, we employ the modified SIMP^43^ (solid isotropic material with penalization) scheme to interpolate the thermal conductivity coefficients of materials 1 and 2, i.e.,$\,{\kappa }_{{{{{{\mathrm{material}}}}}}1}$ and ${\kappa }_{{{{{{\mathrm{material}}}}}}2}$. Specifically, $\kappa ({\rho }_{e})={\kappa }_{{{{{{\mathrm{material}}}}}}1}+{\rho }_{e}^{p}({\kappa }_{{{{{{\mathrm{material}}}}}}2}-{\kappa }_{{{{{{\mathrm{material}}}}}}1})$, where *p* is the penalty coefficient that helps to force the design variables as 0 and 1 in the final optimized design. We set *p* = 5 in topology optimization of TFCs (the influence of *p* is discussed in Supplementary Note 2). To obtain the TFC with the input ${\kappa }_{ilm}^{{{{{{\rm{Input}}}}}}}$, one can formulate a topology optimization model by taking minimizing the difference between the output ${\kappa }_{ilm}^{{{{{{\rm{Output}}}}}}}$ and the input ${\kappa }_{ilm}^{{{{{{\rm{Input}}}}}}}$ as the objective function with the volume fraction of one material as the constraint. However, the selection of the volume fraction of a material is blind in this model, and such objective and constraint functions may cause useless results for some volume fractions^40^ (see more discussions in Supplementary Note 2). To tackle this issue, we transform the objective function into minimizing the volume fraction with the difference between the output ${\kappa }_{ilm}^{{{{{{\rm{Output}}}}}}}$ and the input ${\kappa }_{ilm}^{{{{{{\rm{Input}}}}}}}$ as the constraint reversely, which is formulated as

2 $$\begin{array}{c}\mathop{\min }\limits_{{\rho }_{e}}\,C=\frac{1}{|V|}\mathop{\sum }\limits_{e=1}^{N}{\rho }_{e}\\ s.t.\,:\,{{{{{\bf{K}}}}}}({\rho }_{e}){{{{{\bf{T}}}}}}={{{{{\bf{Q}}}}}}\\ \,G=f\left({\left({\kappa }_{ilm}^{{{{{{\rm{Output}}}}}}}-{\kappa }_{ilm}^{{{{{{\rm{Input}}}}}}}\right)}^{2}\right)=0\\ \,0\le {\rho }_{e}\le 1,\,e=1,{{{{\mathrm{2..}}}}}.N\end{array}$$

where *f*(.) is a continuous function to balance the difference between the output ${\kappa }_{ilm}^{{{{{{\rm{Output}}}}}}}$ and the input ${\kappa }_{ilm}^{{{{{{\rm{Input}}}}}}}$, as $f({({\kappa }_{ilm}^{{{{{{\rm{Output}}}}}}}-{\kappa }_{ilm}^{{{{{{\rm{Input}}}}}}})}^{2})={({\kappa }_{i11}^{{{{{{\rm{Output}}}}}}}-{\kappa }_{i11}^{{{{{{\rm{Input}}}}}}})}^{2}/a+{({\kappa }_{i22}^{{{{{{\rm{Output}}}}}}}-{\kappa }_{i22}^{{{{{{\rm{Input}}}}}}})}^{2}/b+{({\kappa }_{i12}^{{{{{{\rm{Output}}}}}}}-{\kappa }_{i12}^{{{{{{\rm{Input}}}}}}})}^{2}+{({\kappa }_{i21}^{{{{{{\rm{Output}}}}}}}-{\kappa }_{i21}^{{{{{{\rm{Input}}}}}}})}^{2}$, where *a* and *b* are dimensionless and only take the values of ${\kappa }_{i11}^{{{{{{\rm{Input}}}}}}}$ and ${\kappa }_{i22}^{{{{{{\rm{Input}}}}}}}$, respectively. ${{{{{\bf{K}}}}}}({\rho }_{e})$, **T** and **Q** are respectively the global heat conduction matrix, global temperature matrix and global thermal load matrix. ${{{{{\bf{K}}}}}}({\rho }_{e})$ and **Q** can be respectively quantified by ${{{{{\bf{K}}}}}}({\rho }_{e})={\sum }_{e=1}^{N}{{{{{{\bf{k}}}}}}}_{e}$ and ${{{{{\bf{Q}}}}}}={\sum }_{e=1}^{N}{{{{{\bf{N}}}}}}{{{{{{\bf{q}}}}}}}_{e}^{0}$. We use the gradient-based method of moving asymptotes (MMA)^44–46^ to update design variables ${\rho }_{e}$ in Eq. (2). Thus, we calculate sensitivities of constraint *G* and objective function *C* in Eq. (2) by differentiating them with respect to the design variable ${\rho }_{e}$ following the adjoint method^47^. From the topology optimization model in Eq. (2), we can find that when the value of the constraint function *G* is small enough, the optimized TFC can possess the desired equivalent macroscopic thermal conductivity tensor, which keep invariant under different heat flow directions, as presented in Fig. 2(d). This ensures the omnidirectional functionalities of the robustly printable freeform thermal metamaterials. After obtaining all the TFCs by topology optimization as shown in Fig. 2(e), we then assemble these TFCs into freeform thermal metadevices. To promote the interconnection between adjacent TFCs, we fix the four corners in each TFC with material 2 (see Supplementary Note 3 for details). As a result, the metadevices here are in a whole without thermal contact resistance between adjacent TFCs, as seen in Fig. 2(f). Note that our assembling scheme of the freeform thermal metamaterials are different from the traditional ways of assembling with bolts in mechanical metamaterials^48^. Besides, TFCs are freeform and different from each other, and as long as the size of TFCs is small enough, the arbitrary-shape thermal metadevices can be achieved.

### Numerical verifications of omnidirectional thermal functionalities

**Fig. 3:**
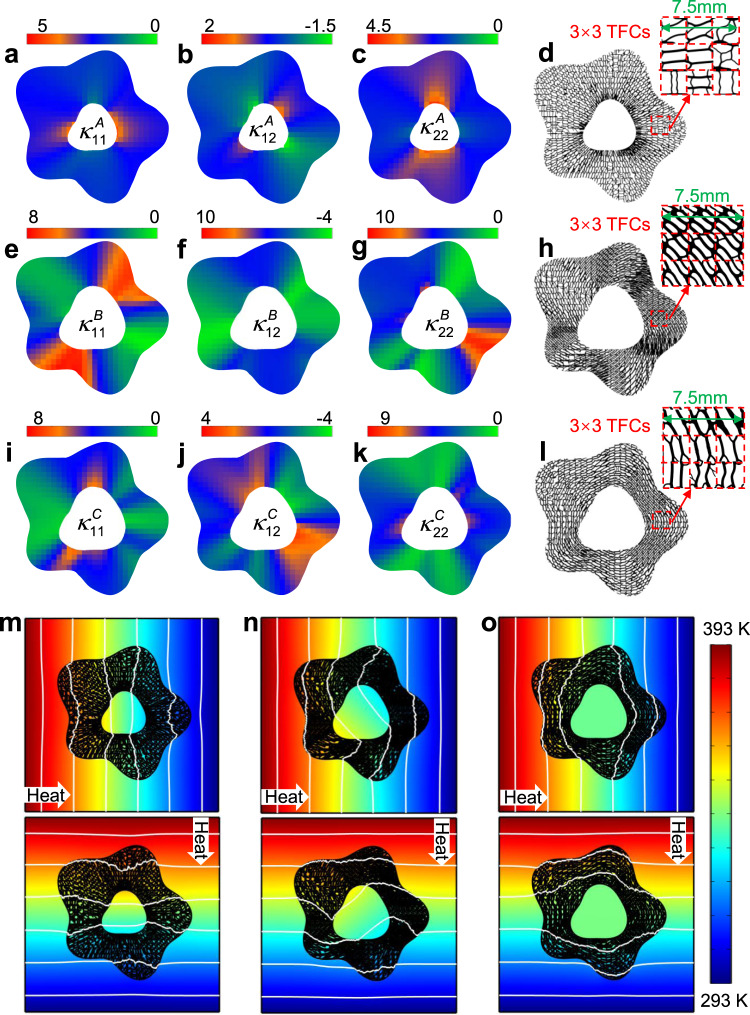
Topological structures of thermal concentrator, rotator, and cloak by assembling TFCs and the corresponding simulated temperature fields. Thermal conductivity tensor distributions of each TFCs in the designed thermal concentrator (**a**–**c**), rotator (**e**–**g**), and cloak (**i**–**k**). Each pixel represents a TFC and its color denotes the value of thermal conductivity. Robustly printable freeform meta-structure of thermal concentrator (**d**), rotator (**h**), and cloak (**l**), respectively. Inside the red dotted box are 3 × 3 TFCs and the size of each TFC is 2.5 mm × 2.5 mm. Simulated temperature fields of thermal concentrator (**m**), rotator (**n**), and cloak (**o**) with the heat flow from left to right and from top to bottom, respectively. The color legend denotes the high and low temperature, and white lines are isothermal lines therein.

To verify the effectiveness of our BT-independent design paradigm, we design arbitrary-shape thermal concentrator, rotator, and cloak, and then evaluate their performance by FEM simulations first. We consider the two-dimensional 100 mm × 100 mm structure with the background thermal conductivity 2.3 Wm^−1^ K^−1^ in Fig. 2(b). Following the above design steps, we obtain three thermal metadevices with detailed parameters in Supplementary Note 4. It is naturally perceived that the smaller the size of each TFC is, the better performance the freeform thermal metamaterials will have. We set the size of TFC as 2.5 mm × 2.5 mm throughout this study and divide the TFC into *N* = 100 × 100 square finite elements (thus the size of each element is 0.025 mm × 0.025 mm) with the balance of the FEM computational efficiency and accuracy. By substituting the parameters into transformation optics theory, the theoretical thermal conductivity tensor distributions of three thermal metadevices are respectively displayed in Fig. 3(a)–(c), (e)–(g), and (i)–(k). We choose two materials—Die steel (H13, ${\kappa }_{{{{{{\rm{H13}}}}}}}$= 31 Wm^−1^ K^−1^) and PDMS (${\kappa }_{{{{{{\mathrm{PDMS}}}}}}}$ = 0.16 Wm^−1^ K^−1^), to calculate the target thermal conductivity tensors and the optimized TFCs. The reason for choosing these two materials is twofold: one is that the prescribed thermal conductivity tensors shown in Fig. 3(a)–(c), (e)–(g), and (i)–(k) are within the Wiener bounds^49^ of the mixture of material H13 and PDMS; and the other is for feasible implementation of 3D printing. The details of simulation verification for a typical TFC are shown in Supplementary Note 5. Moreover, Supplementary Fig. 5 shows the values of constraint function *G* for the three optimized metadevices. It can be seen that the difference between output ${\kappa }_{ilm}^{{{{{{\rm{Output}}}}}}}$ and the input ${\kappa }_{ilm}^{{{{{{\rm{Input}}}}}}}$ are small. After all the TFCs are obtained, we assemble and shape them into freeform thermal concentrator, rotator and cloak, which are shown in Fig. 3(d), (h), and (i), respectively. Besides, the red dotted line boxes, respectively, show the detailed structure of 3 × 3 TFCs, and the connectivity of TFCs in three thermal metadevices is checked in Supplementary Note 3.

To evaluate the omnidirectional thermal functionalities, we impose the same temperature gradient from different directions on the background by setting constant boundary temperatures as *T*_max_ = 393 K and *T*_min_ = 293 K. Besides, to maintain the linear temperature gradient, we set the boundaries parallel to the temperature gradient as adiabatic boundaries. The full wave simulation is conducted by using the FEA simulation software COMSOL Multiphysics 5.5. The simulated temperature distribution of three metadevices are respectively plotted in Fig. 3(m), (n), and (o), with the isothermal lines plotted in white. From Fig. 3(m)–(o), we can see that with these three thermal metadevices, the BT fields are almost not affected after embedding objects in the background since the BT fields remain the same as if the embedded objects are not there, just as illustrated by the ideal temperature fields in Fig. 1(a). While the temperature fields in the object region of three thermal metadevices are concentrated, rotated, and cloaked, respectively. Although we assemble the TFCs in *x*-direction and *y*-direction, the output ${\kappa }_{ilm}^{{{{{{\rm{Output}}}}}}}$ of these TFCs do not change even when the heat flow flows from other directions, validating the omnidirectional thermal functionalities. Further simulations in Supplementary Fig. 6 verify that the thermal metadevices maintain their thermal functionalities well when heat flow is imposed from more different directions, like ±45°. From Fig. 3 and Supplementary Fig. 6, we can see that the proposed freeform thermal metamaterials maintain omnidirectional thermal concentrating, rotating, and cloaking functionalities. In addition, we further study the thermal behavior of three metadevices under non-uniform boundary conditions and transient scenarios, as shown in Supplementary Fig. 7(b). It is obvious that the three thermal metadevices keep their thermal functionalities well, respectively.

### Experimental verifications of omnidirectional thermal functionalities

**Fig. 4:**
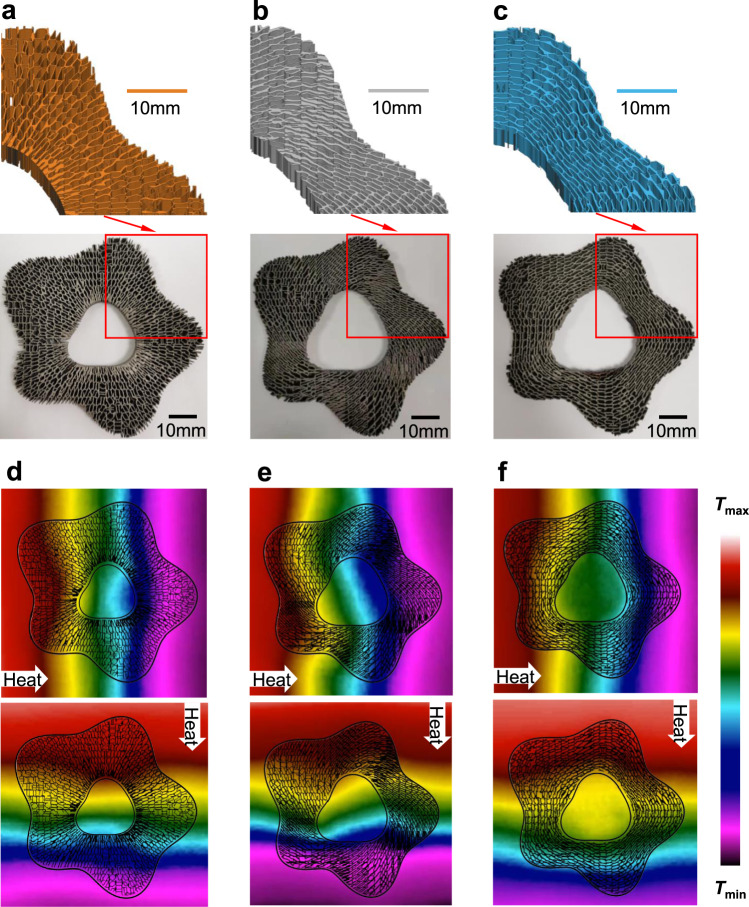
Experimental temperature fields of the three freeform thermal metadevices. **a**–**c** 3D-printed freeform thermal metamaterials of thermal concentrator, rotator, and cloak (without the PVC cover and PDMS fillings). The insets show part of the 3D-printed freeform thermal metadevices. **d**–**f** Experimentally measured temperature fields of the three thermal metadevices for thermal concentrating, rotating, and cloaking, respectively.

For experimental verifications, we fabricate these freeform thermal metadevices by 3D printing with the 5 mm-thick Die steel (H13, 31 Wm^−1^ K^−1^), which can be seen in Fig. 4(a)–(c). Then, we fill the fluidic PDMS into the porous structures (H13) and then solidify the PDMS to fabricate the experimental metadevices. To be consistent with previous designs, we solidify silicone adhesive sealant (ACC AS1802, 2.3 Wm^−1^ K^−1^) as the background medium in a 100 mm × 100 mm × 5 mm acrylic frame with the thermal metadevices embedded. The Peltier heating and cooling modules are fixed at the two ends of the solidified background plate for generating the linear temperature gradient. The experimental setup is schematically drawn in Supplementary Fig. 8(a). Moreover, the whole thermal conductive system is covered with the polyvinyl chloride (PVC) adhesive tape whose thickness is 0.1 mm to maintain the same surface emissivity in the infrared camera.

After the temperature of the Peltier heating and cooling modules becomes steady, we put the infrared camera (SEEK Compact PRO) vertically above the thermal metadevices to measure the temperature field. As shown in Fig. 4(d)–(f), it is seen that the external temperature field of the background is basically not affected by the thermal metadevices, while the temperature fields in the object region show the thermal concentrating, rotating, and cloaking effects, respectively. From both the experimental (Fig. 4(d)–(f)) and simulated (Fig. 3(m)–(o)) temperature fields, we can see clearly the three corresponding thermal functionalities as thermal concentrating, rotating, and cloaking, which are further validated through the quantitative comparison between the simulated and experimental temperature fields in Supplementary Fig. 8(b)–(d). Therefore, it is concluded that via the numerical and experimental verifications, the present robustly printable freeform thermal metamaterials are effective for designing arbitrary-shape thermal metadevices, including but not limited to those examples in this work.

In summary, we propose a BT-independent design paradigm for robustly printable freeform thermal metamaterials that can overcome the three long-standing challenges of traditional thermal metamaterials. We numerically and experimentally demonstrate that the robustly printable freeform thermal metamaterials (thermal concentrator, rotator and cloak) are effective on manipulating the heat flow omnidirectionally. Our study presents breakthroughs in the realization of robust, powerful and assembled thermal metamaterials, and we believe our 3D-printing-assisted recipe may trigger more investigations into robust thermal functionalities. Moreover, it is convenient to extend the 2D thermal metamaterials into 3D counterparts by tailoring mathematical models of topology optimization. It is expected that robustly printable freeform thermal metamaterials may be coupled with moving, dynamic or intelligent materials^12,50,51^, to achieve more powerful thermal metamaterials.

## Methods

### Details in design and assembly of TFCs

The details in design and assembly of TFCs into freeform thermal metamaterials are shown in Supplementary Fig. 9. To maintain material connection, we fix the four corners of each TFC filled with material 2 to promote that the adjacent TFCs can be connected as a whole structure. For topology optimization of a TFC, the initial and optimized structures are shown in Supplementary Fig. 9(a, b), respectively. Due to the utilization of the SIMP method, the optimized TFC has intermediate density elements and fuzzy boundaries. Then, the boundaries of the optimized TFC are obtained by the binarization of the density with the threshold 0.5. The process and the smoothed TFC structure are shown in Supplementary Fig. 9(c). Next, all TFCs are assembled, which is shown in Supplementary Fig. 9(d). Besides, we need to remove the redundant material caused by the assembly. Supplementary Fig. 9(e) gives the final thermal meta-structure for a concentrator. In above steps, MATLAB R2017a codes are written to obtain the optimized TFCs and assembled thermal metamaterials.

### Numerical modeling

The thermal functionalities of robustly printable freeform thermal metamaterials are verified by the FEA simulation commercial software COMSOL Multiphysics 5.5. To ensure the model consistency, the interface COMSOL Multiphysics 5.5 with MATLAB R2017a is used to create the simulated model. In COMSOL Multiphysics 5.5, about 3.5 million triangular meshes are used to divide the simulated region freely. Later, the boundary conditions are imposed and the free solver in COMSOL Multiphysics 5.5 is used to solve the steady-state temperature field distribution which can be seen in Fig. 3(m)–(o). For transient cases, the densities and heat capacities of the two materials are set as: H13 (*ρ* = 7850 kg m^−3^, *c*_*p*_ = 650 J kg^−1^ K^−1^); PDMS (*ρ* = 970 kg m^−3^, *c*_*p*_ = 1460 J kg^−1^ K^−1^); ACC AS1802 (*ρ* = 1060 kg m^−3^, *c*_*p*_ = 1615 J kg^−1^ K^−1^). The simulated results of transient cases are shown in Supplementary Fig. 7(a).

### Generation of STL model for thermal metamaterials

The STL model for 3D printing is directly generated from the output of MATLAB R2017a. Then, it is imported into the commercial software Materialise Magics 24.0. In Materialise Magics 24.0, the STL model is scaled to the design size and repaired by the automatic repair function. Finally, we can obtain the STL models in the top half of Fig. 4(a)–(c).

## Supplementary information

Supplementary information.

## Data Availability

The STL files of three thermal metadevices for 3D printing are available at 10.6084/m9.figshare.16831969. Additional data that support the findings of this study are available from the corresponding authors upon reasonable request.

## Code availability

All codes necessary to reproduce the results in main paper are available from the corresponding authors upon reasonable request.
