# Supplementary material for: Robustly printable freeform thermal metamaterials
Source: Nat Commun. 2021 Dec 10;12:7228. doi: 10.1038/s41467-021-27543-7 (PMC8664938; doi:10.1038/s41467-021-27543-7)
Supplement: Supplementary file 1 — Supplementary information. [file 41467_2021_27543_MOESM1_ESM.pdf]

Supplementary Information

Robustly printable freeform thermal metamaterials

Wei Sha[1,a], Mi Xiao[1,a], Jinhao Zhang[1], Xuecheng Ren[2], Zhan Zhu[2], Yan Zhang[1], Guoqiang Xu[3], Huagen Li[3], Xiliang Liu[1], Xia Chen[4], Liang Gao[1,*], Cheng-Wei Qiu[3,†], Run Hu[2,‡]

[1]*State Key Laboratory of Digital Manufacturing Equipment and Technology, Huazhong University of Science and Technology, Wuhan, 430074, China*

[2]*State Key Laboratory of Coal Combustion, School of Energy and Power Engineering, Huazhong University of Science and Technology, Wuhan, 430074, China*

[3]*Department of Electrical and Computer Engineering, National University of Singapore, Kent Ridge 117583, Republic of Singapore*

[4]*School of Electrical and Electronic Engineering, Huazhong University of Science and Technology, Wuhan, 430074, China*

[a]*These authors contributed equally.*

**Email: gaoliang@mail.hust.edu.cn*

[†]*Email: chengwei.qiu@nus.edu.sg*

[‡]*Email: hurun@hust.edu.cn*

Supplementary Note 1

The thermal conductivity tensor calculated by transformation optics

For thermal concentrator, as shown in Fig. 2(a) and (b) of main article, we compress the region $r \le R_3(\theta)$ into the one $r' \le R_1(\theta')$ and extend the region $R_3(\theta) \le r \le R_2(\theta)$ to the one $R_1(\theta') \le r' \le R_2(\theta')$. The geometric transformation formulas are as follows:

(1) in the inner core $r' \le R_1(\theta')$:

$$r' = r\frac{R_1(\theta)}{R_3(\theta)},\ \theta' = \theta \qquad \text{S.(1)}$$

and (2) in the coating region $R_1(\theta') \le r' \le R_2(\theta')$:

$$r' = r\frac{R_2(\theta) - R_3(\theta)}{R_2(\theta) - R_1(\theta)} + \frac{R_2(\theta)(R_1(\theta) - R_3(\theta))}{R_2(\theta) - R_3(\theta)},\ \theta' = \theta \qquad \text{S.(2)}$$

After the algebraic operation, we can obtain the thermal conductivity tensor in Cartesian

coordinate system. In the inner core $r' \le R_1(\theta')$, the thermal conductivity tensor is computed by

$$\boldsymbol{\kappa}^{A_1} = \mathbf{R}(\theta')\begin{bmatrix} (\kappa_{11}^{A_1})_{r\theta} & (\kappa_{12}^{A_1})_{r\theta} \\ (\kappa_{21}^{A_1})_{r\theta} & (\kappa_{22}^{A_1})_{r\theta} \end{bmatrix}\mathbf{R}(\theta')^T \kappa_b = \begin{bmatrix} \kappa_{11}^{A_1} & \kappa_{12}^{A_1} \\ \kappa_{21}^{A_1} & \kappa_{22}^{A_1} \end{bmatrix}$$

$$(\kappa_{11}^{A_1})_{r\theta} = \frac{r'^2 + A_1^{\ 2}}{r'^2}; (\kappa_{12}^{A_1})_{r\theta} = (\kappa_{21}^{A_1})_{r\theta} = \frac{A_1}{r'}; (\kappa_{22}^{A_1})_{r\theta} = 1 \qquad \text{S.(3)}$$

$$A_1 = \frac{r\left(R_2(\theta')\dfrac{dR_1(\theta')}{d\theta'} - R_1(\theta')\dfrac{dR_2(\theta')}{d\theta'} \right)}{R_2(\theta')R_1(\theta')}$$

where $\mathbf{R}(\varphi) = \begin{bmatrix} \cos\varphi & -\sin\varphi \\ \sin\varphi & \cos\varphi \end{bmatrix}$ is the rotational matrix.

In the coating region $R_1(\theta') \le r' \le R_2(\theta')$, the thermal conductivity tensor is computed by

$$\boldsymbol{\kappa}^{A_2} = \mathbf{R}(\theta')\begin{bmatrix} (\kappa_{11}^{A_2})_{r\theta} & (\kappa_{12}^{A_2})_{r\theta} \\ (\kappa_{21}^{A_2})_{r\theta} & (\kappa_{22}^{A_2})_{r\theta} \end{bmatrix}\mathbf{R}(\theta')^T \kappa_b = \begin{bmatrix} \kappa_{11}^{A} & \kappa_{12}^{A} \\ \kappa_{21}^{A} & \kappa_{22}^{A} \end{bmatrix}$$

$$(\kappa_{11}^{A_2})_{r\theta} = \frac{(r'-b)^2 + A_2^{\ 2}}{r'(r'-b)}; (\kappa_{12}^{A_2})_{r\theta} = (\kappa_{21}^{A_2})_{r\theta} = \frac{A_2}{r'-b}; (\kappa_{22}^{A_2})_{r\theta} = \frac{r'}{r'-b}$$

$$A_2 = \frac{(R_2(\theta') - R_1(\theta'))(r' - R_2(\theta'))\dfrac{dR_3(\theta')}{d\theta'}}{(R_3(\theta') - R_1(\theta'))^2} + \qquad \text{S.(4)}$$

$$\frac{(R_3(\theta') - R_1(\theta'))(r' - R_1(\theta'))\dfrac{dR_2(\theta')}{d\theta'} - (R_2(\theta') - R_3(\theta'))(r' - R_2(\theta'))\dfrac{dR_1(\theta')}{d\theta'}}{(R_3(\theta') - R_1(\theta'))^2}$$

$$b = \frac{R_2(\theta)(R_1(\theta) - R_3(\theta))}{R_2(\theta) - R_3(\theta)}$$

For thermal rotator, we employ the following geometric mapping:

$$r' = r, \ \theta' = \theta + \theta_0 \frac{f\left(R_2(\theta)\right) - f\left(r\right)}{f\left(R_2(\theta)\right) - f\left(R_1(\theta)\right)} \qquad \text{S.(5)}$$

Using this transformation, the space rotation is achieved with the change of r. Then, in the region $R_1(\theta') \le r' \le R_2(\theta')$, the thermal conductivity tensor in Cartesian coordinate system can be calculated by

$$\boldsymbol{\kappa}^B = \mathbf{R}(\theta')\begin{bmatrix} (\kappa_{11}^B)_{r\theta} & (\kappa_{12}^B)_{r\theta} \\ (\kappa_{21}^B)_{r\theta} & (\kappa_{22}^B)_{r\theta} \end{bmatrix}\mathbf{R}(\theta')^T \kappa_b = \begin{bmatrix} \kappa_{11}^B & \kappa_{12}^B \\ \kappa_{21}^B & \kappa_{22}^B \end{bmatrix}$$

$$(\kappa_{11}^B)_{r\theta} = \frac{1}{B_1}; (\kappa_{12}^B)_{r\theta} = (\kappa_{21}^B)_{r\theta} = \frac{B_2 r}{B_1}; (\kappa_{22}^B)_{r\theta} = B_1 + \frac{{B_2}^2 r^2}{B_1}$$

$$B_1 = 1 + \theta_0 \frac{\frac{df\left(R_2(\theta)\right)}{d\theta}\left[f\left(R_2(\theta)\right) - f\left(R_1(\theta)\right)\right] - \left[\frac{df\left(R_2(\theta)\right)}{d\theta} - \frac{df\left(R_1(\theta)\right)}{d\theta}\right]\left[f\left(R_2(\theta)\right) - f\left(r\right)\right]}{\left[f\left(R_2(\theta)\right) - f\left(R_1(\theta)\right)\right]^2}$$

$$B_2 = \theta_0 \frac{\frac{df(r)}{d\theta}}{f\left(R_2(\theta)\right) - f\left(R_1(\theta)\right)}$$

S.(6)

where $f(r)$ could be any continuous function of r.

For thermal cloak, we map the region $r \le R_2(\theta)$ into the annular one $R_1(\theta') \le r' \le R_2(\theta')$, the geometric transformation formula is:

$$r' = r\frac{R_2(\theta) - R_1(\theta)}{R_2(\theta)} + R_1(\theta),\ \theta' = \theta \qquad \text{S.(7)}$$

Then, the thermal conductivity tensor in Cartesian coordinate system for the region $R_1(\theta') \le r' \le R_2(\theta')$ can be calculated by

$$\boldsymbol{\kappa}^C = \mathbf{R}(\theta')\begin{bmatrix} (\kappa_{11}^C)_{r\theta} & (\kappa_{12}^C)_{r\theta} \\ (\kappa_{21}^C)_{r\theta} & (\kappa_{22}^C)_{r\theta} \end{bmatrix}\mathbf{R}(\theta')^T \kappa_b = \begin{bmatrix} \kappa_{11}^C & \kappa_{12}^C \\ \kappa_{21}^C & \kappa_{22}^C \end{bmatrix}$$

$$(\kappa_{11}^C)_{r\theta} = \frac{(r' - R_1(\theta'))^2 + C^2}{r'(r' - R_1(\theta'))}; (\kappa_{12}^C)_{r\theta} = (\kappa_{21}^C)_{r\theta} = \frac{C}{r' - R_1(\theta')}; (\kappa_{22}^C)_{r\theta} = \frac{r'}{r' - R_1(\theta')} \qquad \text{S.(8)}$$

$$C = \frac{R_1(\theta')(r' - R_1(\theta'))\frac{dR_2(\theta')}{d\theta'} - R_2(\theta')(r' - R_2(\theta'))\frac{dR_1(\theta')}{d\theta'}}{R_2(\theta')(R_2(\theta') - R_1(\theta'))}$$

Supplementary Note 2

Discussion about the formulation of topology optimization model

In the main article, the goal of topology optimization is to obtain the structure with the target thermal conductivity tensor. Thus, we set the objective function as the difference between the homogenized thermal conductivity tensor and the target one. Then, the topology optimization model is formulated as

$$
\begin{aligned}
&\min_{\rho_e} \; G = f\left((\kappa_{ilm}^{\text{Output}} - \kappa_{ilm}^{\text{Input}})^2\right) \\
&s.t.: \mathbf{K}(\rho_e)\mathbf{T} = \mathbf{Q} \\
&\qquad C = \frac{1}{|V|}\sum_{e=1}^{N} \rho_e \le V_s \\
&\qquad 0 \le \rho_e \le 1, \; e = 1, 2 \ldots N
\end{aligned}
\qquad \text{S.(9)}
$$

where V_s is the volume constraint of material 2. The meanings of the other symbols are the same as those in the main article, and the penalty coefficient p is set as 5 here. Based on this model, the optimized topological functional cell (TFC) structure may be filled with a large number of intermediate densities. Sometimes, we even fail to obtain the optimized structure with the desired thermal conductivity tensor. We take the target thermal conductivity tensor $\boldsymbol{\kappa}_i = \begin{bmatrix} 3.8782 & 0.7144 \\ 0.7144 & 1.4957 \end{bmatrix}$ $\text{Wm}^{-1}\text{K}^{-1}$ as an example to give a detailed explanation as follows:

When a large volume fraction V_s is set, such as 0.6, the superfluous materials tend to prevent the optimization process and in turn produce structures with lots of grey elements, as shown in Supplementary Figure 1(a). When a small volume fraction V_s is set, such as 0.1, we will not be able to obtain the structure with desired thermal conductivity tensor, as shown in Supplementary Figure 1(b). When adjusting the volume fraction $V_s = 0.2$, we obtain the optimized structure in Supplementary Figure 1(c), where the effective thermal conductivity of the optimized structure is close to the target one. Therefore, for the topology optimization model in Eq. S.(9), the selection of the volume fraction V_s is blind, which may cause the useless results.

In contrast, we use the topology optimization model in Eq. (2) of main article, where we minimize the volume fraction V_s and the difference between the homogenized thermal conductivity tensor and the target one is considered in a constraint. We can conveniently obtain the optimized structure with the desired thermal conductivity tensor without artificial selecting and adjusting the volume fraction V_s, which can be reflected in Supplementary Figure 2(a). Besides, we tune the penalty coefficient p from 3 to 5, and the optimized structures are shown in Supplementary Figure 2. Obviously, it can be seen that when p = 3 or 4, too many grey elements exist in the optimized

structures. While the optimized structure with less intermediate materials can be obtained when p = 5.

Supplementary Note 3

The connectivity of TFCs

To promote the connectivity of TFCs in designing thermal metamaterials, we fix the four corners of each TFC as material H13. Sometimes the discontinuous phenomenon may occur between the material fixed at the corners and the one distributed in the other region within the TFC, as shown in Supplementary Figure 3(b). Nevertheless, this phenomenon happens very rarely. Statistically, there are 1560 TFCs in the designed metadevices, and only for 18 TFCs, the above phenomenon occurs. For these 18 TFCs (some of them can be seen in the insets of Supplementary Figure 3(a)), although the connectivity does not occur at the fixed four corners, they have a good connectivity with the surrounding TFCs. Therefore, these few TFCs still can connect with their adjacent TFCs well in the final thermal metamaterials. If they cannot connect with the surrounding TFCs, we will re-optimize to promote the connectivity under fixing other positions within the TFC design area as material H13.

Supplementary Note 4

Parameter setting for three thermal metadevices in the numerical simulation

The parameters in numerical simulation for three thermal metadevices are set as follows:

(1) for thermal concentrator,

$$\begin{aligned} R_2(\theta') &= \left[15+\sin(\theta')-\sin(2\theta')+2\cos(5\theta')\right]/0.48\,\text{mm} \\ R_1(\theta') &= 0.8*\left[17+2\cos(\theta')+\sin(2\theta')-2\sin(3\theta')\right]/1.2\text{mm} \\ R_3(\theta') &= 1.2*\left[17+2\cos(\theta')+\sin(2\theta')-2\sin(3\theta')\right]/1.2\,\text{mm} \end{aligned} \qquad \text{S.(10)}$$

(2) for thermal rotator,

$$\begin{aligned} \theta_0 &= \frac{\pi}{4} \\ R_2(\theta') &= \left[15+\sin(\theta')-\sin(2\theta')+2\cos(5\theta')\right]/0.48\,\text{mm} \\ R_1(\theta') &= \left[17+2\cos(\theta')+\sin(2\theta')-2\sin(3\theta')\right]/1.2\text{mm} \end{aligned} \qquad \text{S.(11)}$$

and (3) for thermal cloak,

$$R_2(\theta') = \left[15 + \sin(\theta') - \sin(2\theta') + 2\cos(5\theta')\right] / 0.48\,\text{mm}$$
$$R_1(\theta') = \left[17 + 2\cos(\theta') + \sin(2\theta') - 2\sin(3\theta')\right] / 1.2\text{mm} \qquad \text{S.(12)}$$

Supplementary Note 5

Numerical verifications for TFC

Here, we evaluate the homogenized thermal conductivity tensor $\kappa_{ilm}^{\text{Output}}$ by comparing the temperature field distribution under the same boundary conditions for two different cases.

We take the thermal conductivity tensor $\boldsymbol{\kappa}_i = \begin{bmatrix} 4.1807 & -1.5417 \\ -1.5417 & 1.8339 \end{bmatrix}$ $Wm^{-1}K^{-1}$ as an example. Case 1 is that the temperature field distribution of a pure plate with the given target thermal conductivity is calculated by software COMSOL Multiphysics 5.5. Firstly, we construct a 12.5 mm×12.5 mm square plate, whose thermal conductivity is set as the target one, i.e., $\boldsymbol{\kappa}_i = \begin{bmatrix} 4.1807 & -1.5417 \\ -1.5417 & 1.8339 \end{bmatrix}$ $Wm^{-1}K^{-1}$. Then, we impose temperature gradient on the pure plate by setting boundary temperatures as $T_{\max}$ = 393 K and $T_{\min}$ = 293 K. To maintain the linear temperature gradient, we set the boundaries parallel to the temperature gradient as adiabatic boundaries. Finally, the simulated temperature field distribution is displayed in Supplementary Figure 4(e).

Case 2 is that the temperature field distribution of the reprocessed TFC with the two materials is calculated by software COMSOL Multiphysics 5.5. After topology optimization, we obtain the optimized TFC shown in Supplementary Figure 4(b) and the reprocessed TFC is shown in Supplementary Figure 4(c). Then, based on the reprocessed TFC, we construct a 5×5 periodic structure (black represents H13 and white represents polydimethylsiloxane PDMS in Supplementary Figure 4(c)), and its size is 12.5 mm×12.5 mm as well. The thermal conductivities of two materials are set as 31 $Wm^{-1}K^{-1}$ and 0.16 $Wm^{-1}K^{-1}$, respectively. Finally, the simulated temperature field distribution is calculated under the same boundary conditions as Supplementary Figure 4(e) and the result is shown in Supplementary Figure 4(d).

The simulation in Supplementary Figure 4(e) is performed based on the theoretical value $\boldsymbol{\kappa}_i = \begin{bmatrix} 4.1807 & -1.5417 \\ -1.5417 & 1.8339 \end{bmatrix}$ $Wm^{-1}K^{-1}$. Thus, the temperature field distribution in Supplementary Figure 4(e) can be considered as the reference one. To quantitatively compare temperature field distributions, we calculate the temperature values of the points on the observation lines (i.e., the black dotted line in Supplementary Figure 4(d) and the violet solid line in Supplementary Figure 4(e)), as shown in Supplementary Figure 4(f). The temperature values obtained by the structural simulation (Case 2) is almost equal to that obtained by the theoretical simulation (Case 1). Therefore, it is illustrated that the macroscopic equivalent thermal conductivity tensor of the reprocessed TFC is very close to the desired one $\boldsymbol{\kappa}_i$.

Supplementary Figure

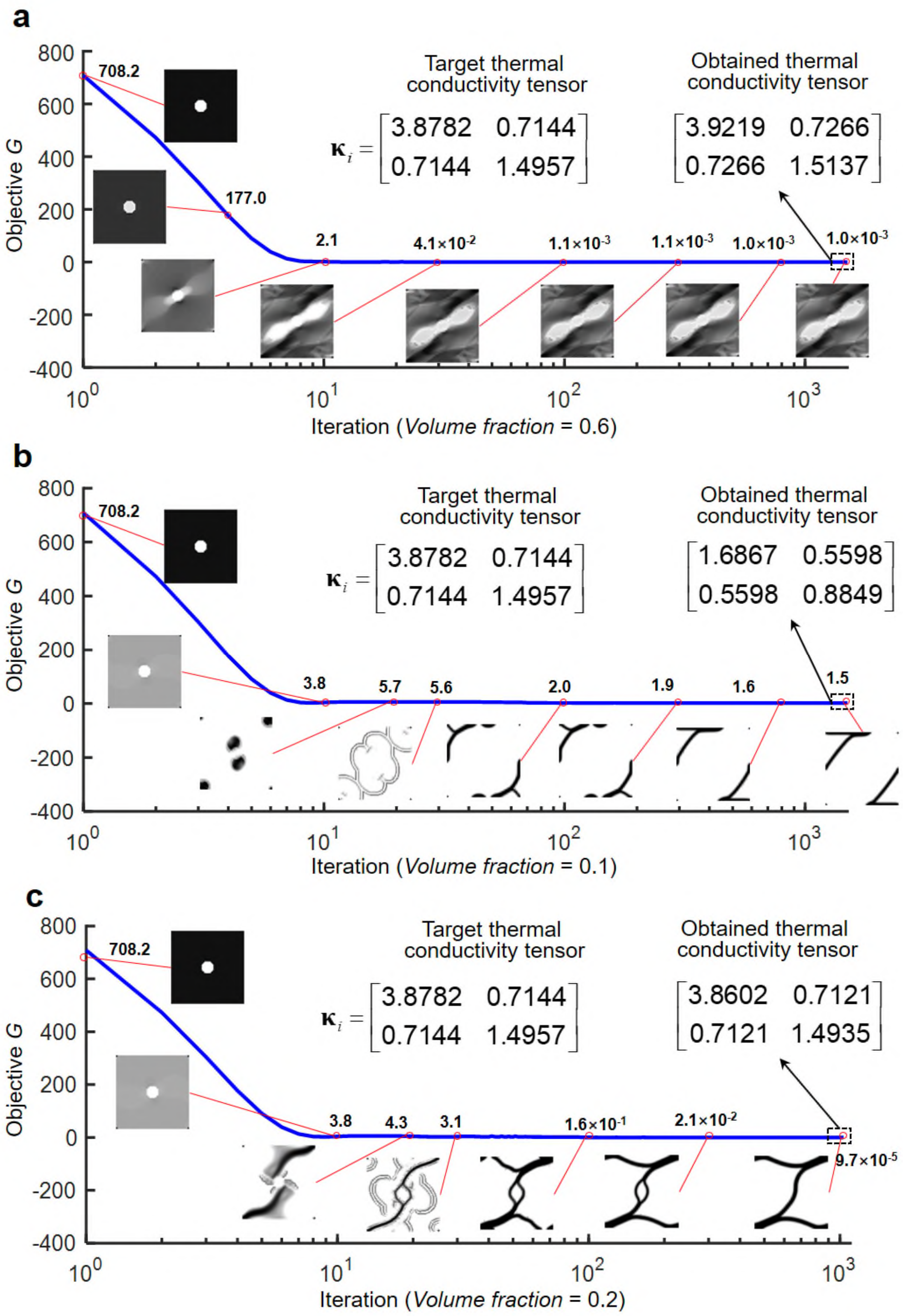

Supplementary Figure 1. Optimization process based on topology optimization model in Eq. S.(13) with several intermediate designs. (a)-(c) V_s = 0.6, 0.1 and 0.2. The horizontal and vertical coordinates represent the number of iterations and the value of the objective function G, respectively.

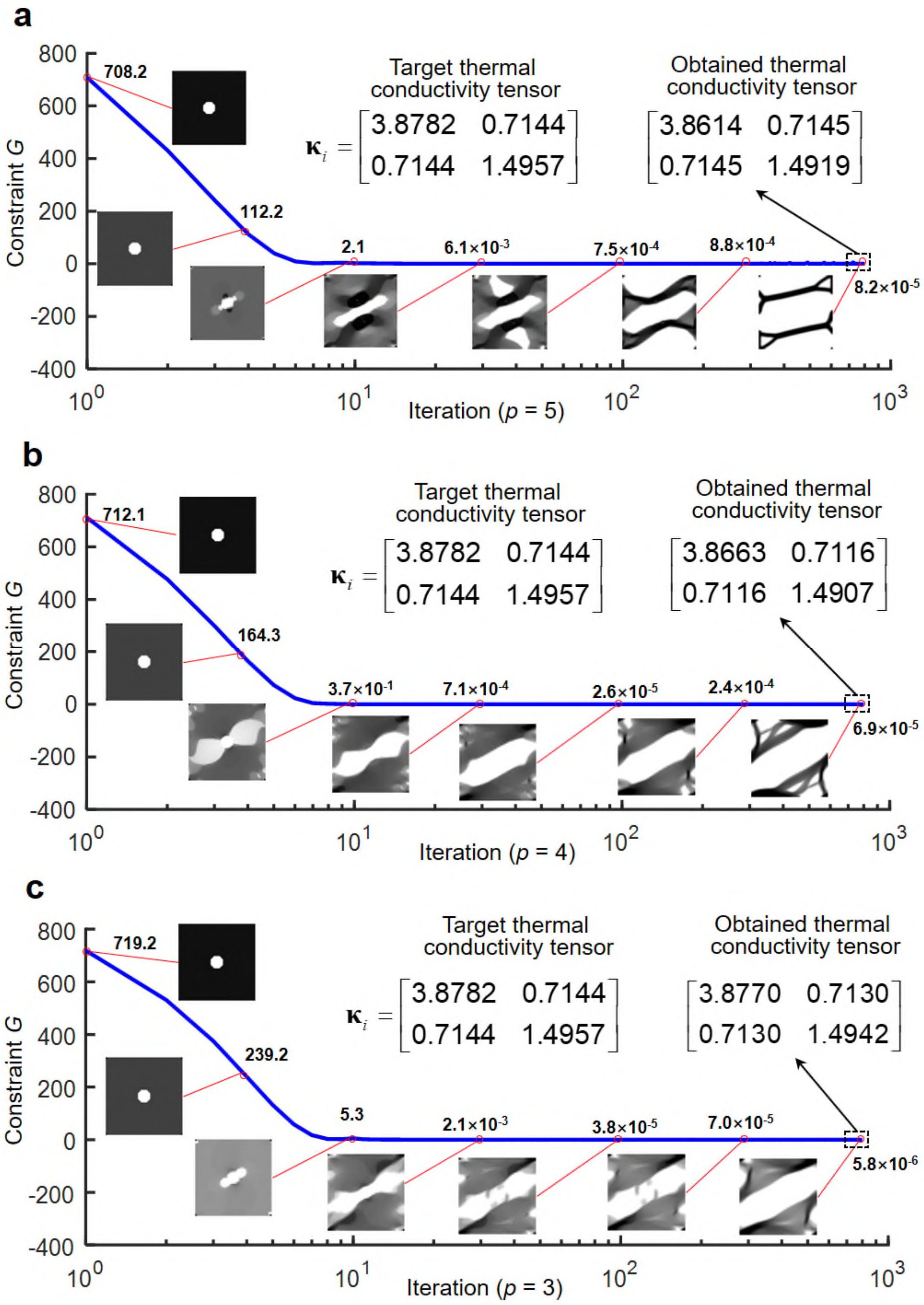

Supplementary Figure 2. Optimization process based on topology optimization model in Eq. (2) of main article with several intermediate designs. (a)-(c) $p = 5$, 4 and 3. The horizontal and vertical coordinates represent the number of iterations and the value of the constraint function G, respectively.

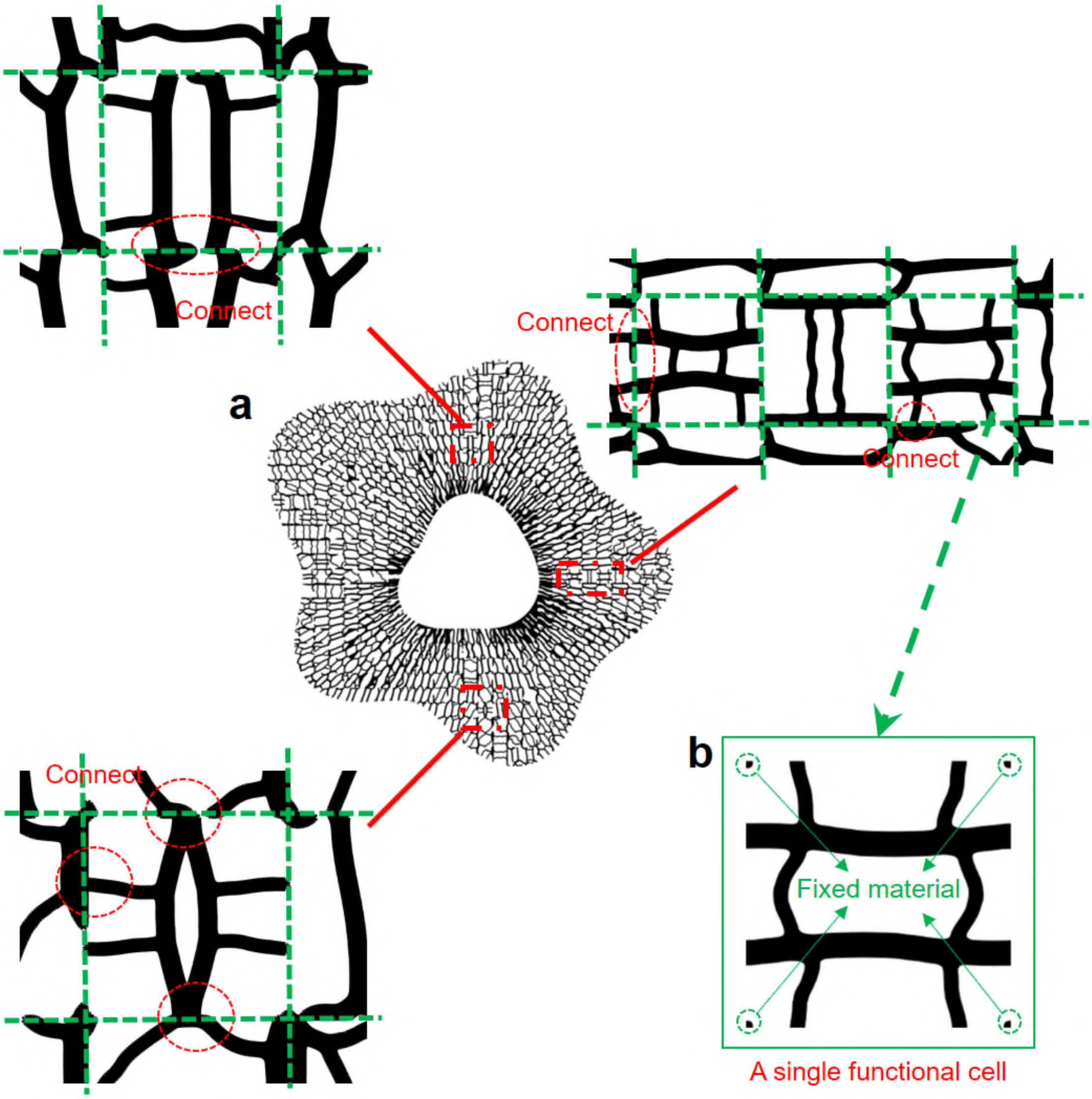

Supplementary Figure 3. The connectivity of TFCs in thermal concentrator. (a) Robustly printable freeform meta-structure of thermal concentrator. The insets show the partial enlarged details of the thermal concentrator. **(b)** A single TFC that the material fixed at the corners and the one distributed in the other region within the TFC are discontinuous, but connects with the surrounding TFCs.

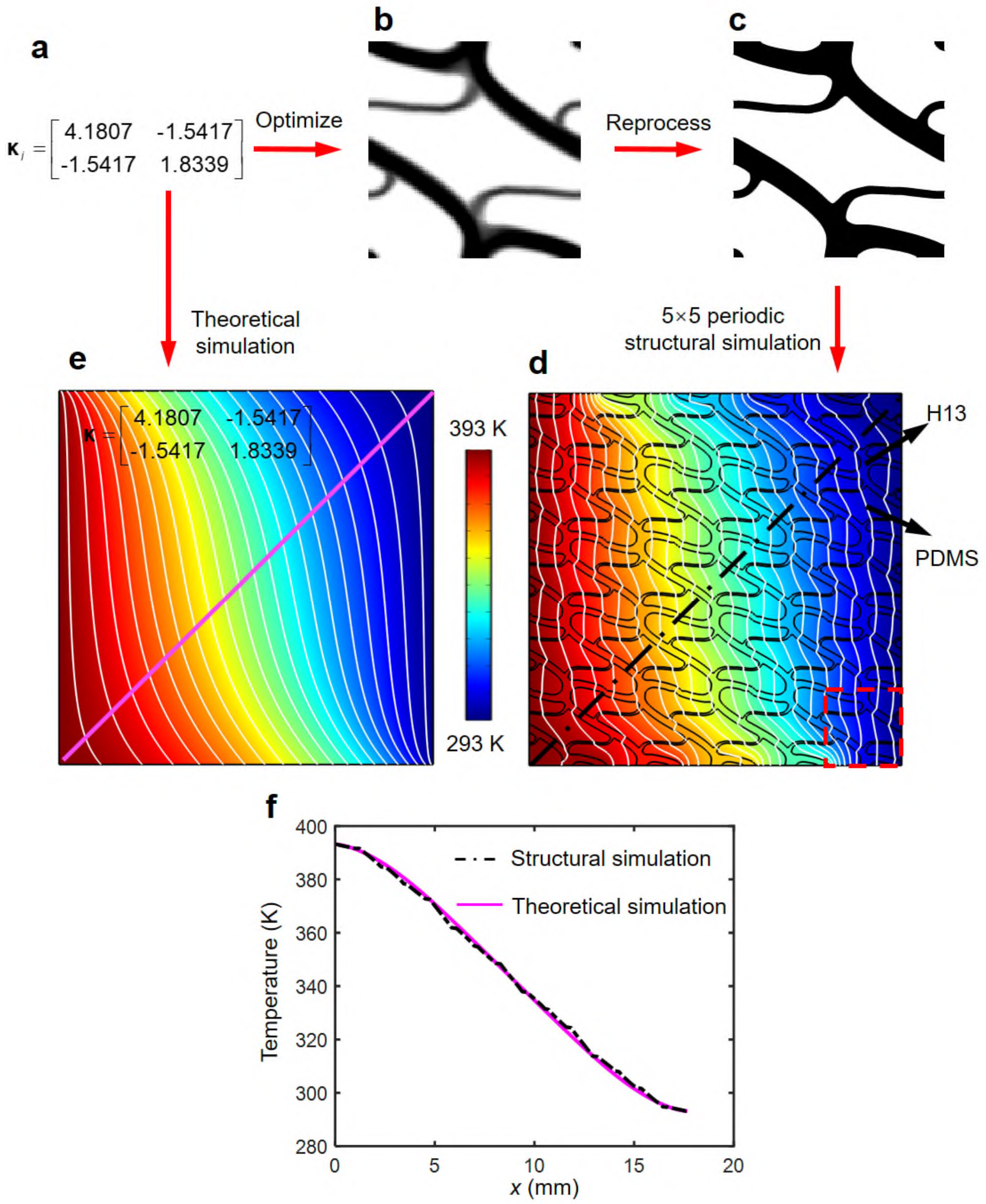

Supplementary Figure 4. Numerical verifications for the TFC. (a) The desired thermal conductivity tensor $\boldsymbol{\kappa}_i$. **(b)** Optimized structure of a TFC. **(c)** Reprocessed structure of the TFC. **(d)**, **(e)** Temperature distribution of the 5×5 periodic structures and an anisotropy plate with a horizontal heat flow, respectively. The black dotted and violet lines are observational lines. Red dashed lines in **(d)** are a single reprocessed structure. Isothermal lines are represented with white color in **(d)** and **(e)**. **(f)** Temperature along the observational lines in **(d)** and **(e).**

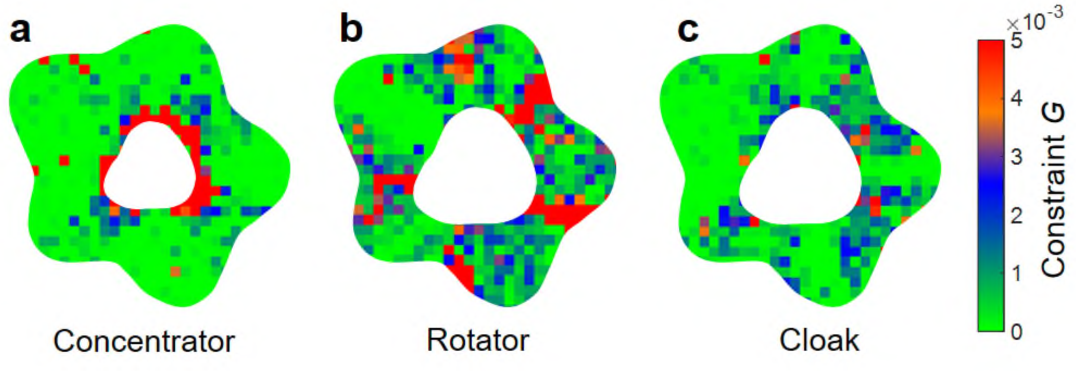

Supplementary Figure 5. Values of constraint function G for three optimized metadevices, i.e., the errors between the target and obtained effective thermal conductivity tensors for **(a)** concentrator, **(b)** Rotator and **(c)** Cloak. Each pixel represents a TFC and its color denotes the value of G.

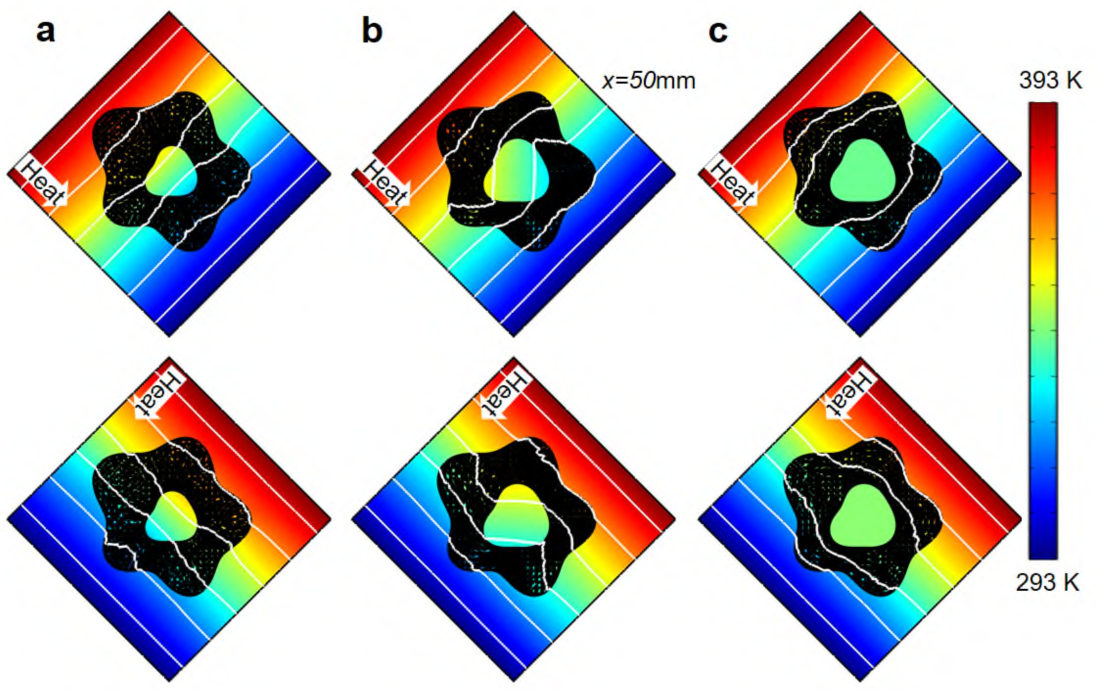

Supplementary Figure 6. Numerical verifications of three thermal metadevices with different heat flow. (a)-(c): The temperature distribution of thermal concentrator, thermal rotator and thermal cloak with 45° and 135° downward heat flows. The white lines are isotherms.

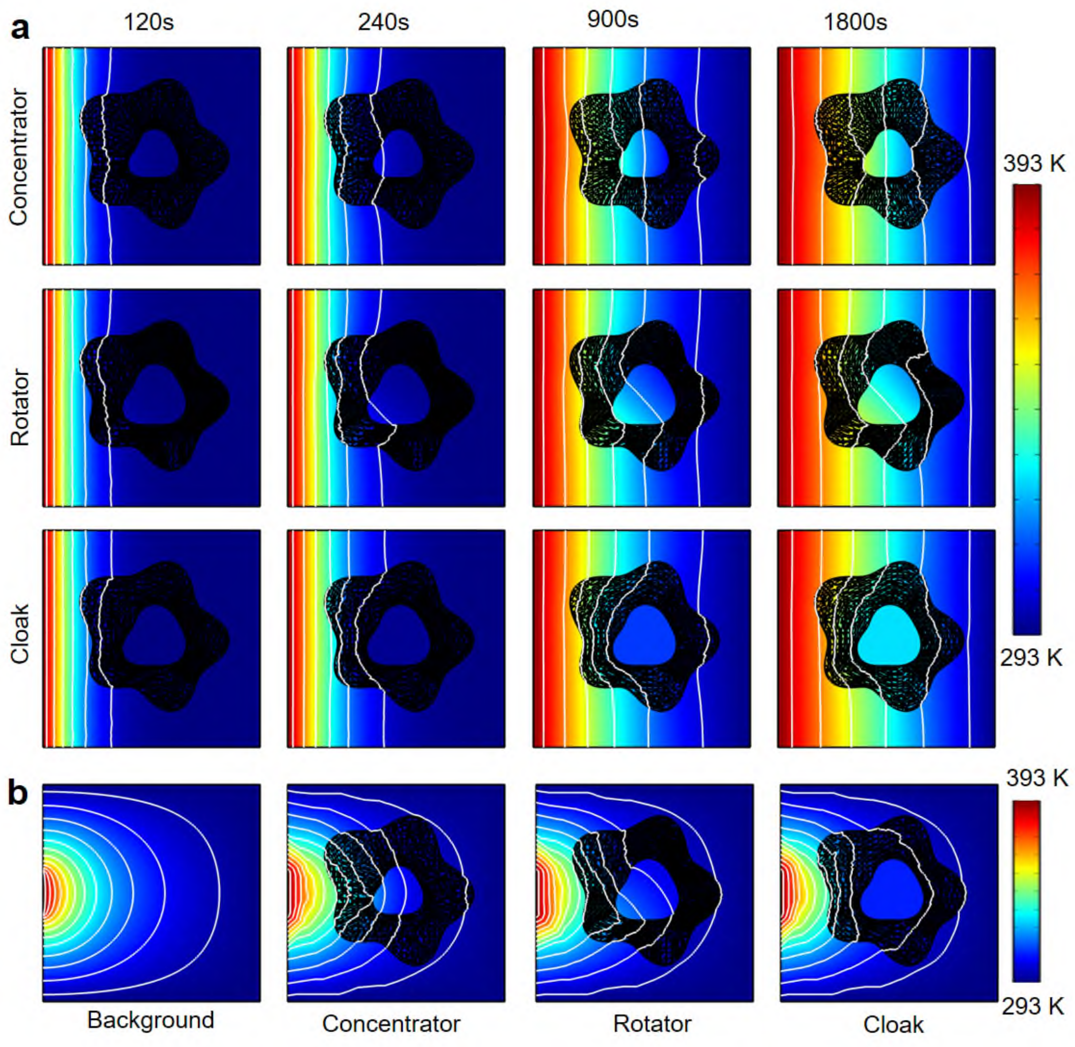

Supplementary Figure 7. Numerical verifications of three thermal metadevices under transient case and non-uniform thermal field. (a) Transient temperature distribution of thermal concentrator, rotator and cloak at different times *t* =120s, 240s, 900s and 1800s. **(b)** Temperature distribution of uniform material, thermal concentrator, rotator and cloak under the non-uniform boundary condition. The white lines are isotherms.

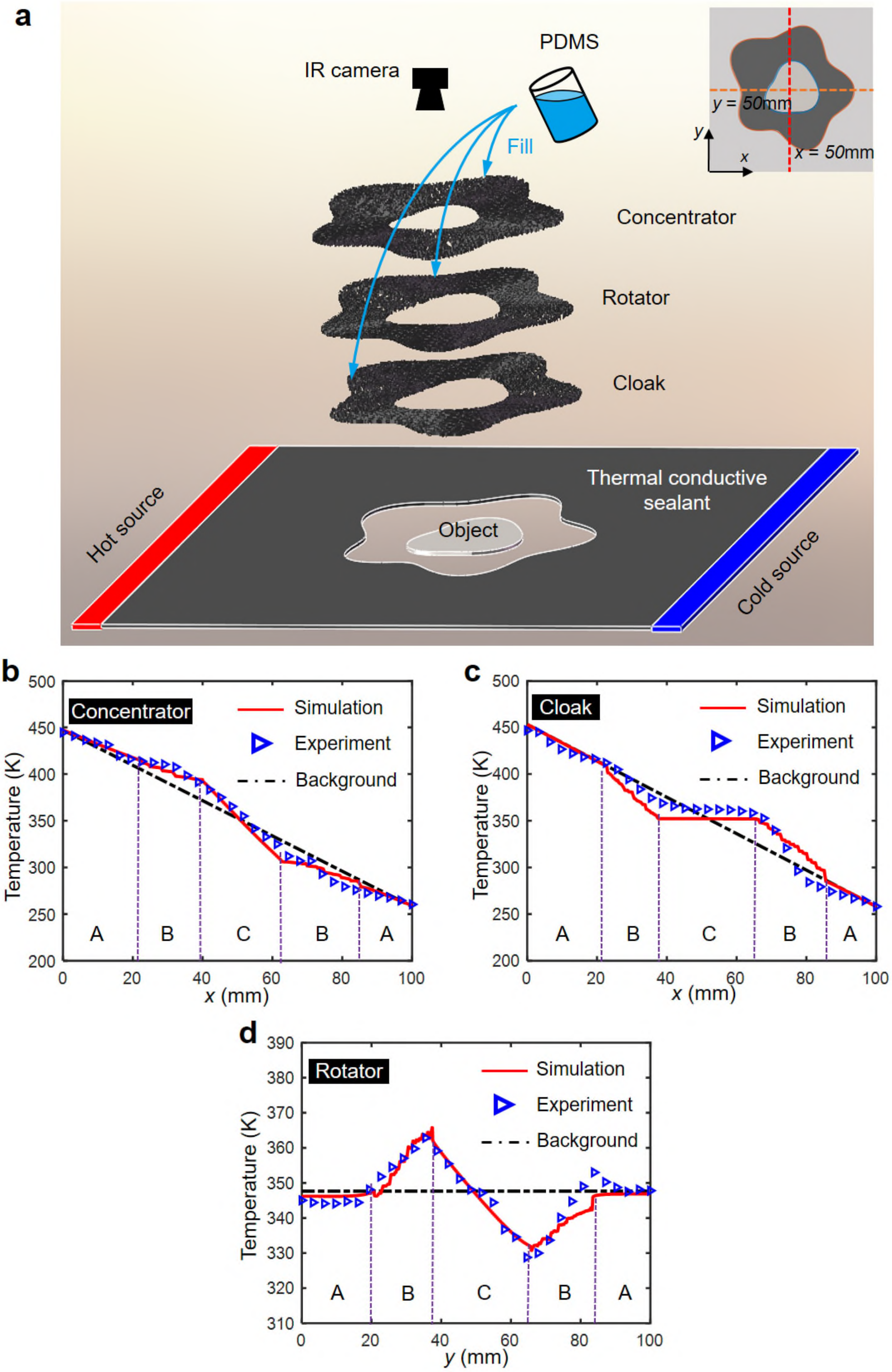

Supplementary Figure 8. Experimental verifications of three thermal metadevices under uniform temperature gradient. (a) Experimental setups of the demonstrated scheme. The inset shows the section lines for calculation of experimental temperature field. **(b), (c)** Experimental

calculated temperature points for thermal concentrator and cloak at gold observational line in inset of **(a). (d)** Experimental calculated temperature points for thermal rotator at red observational line in inset of **(a).** The temperature markers in **(b)-(d)** are calculated from the surface temperature data collected by the IR camera. The A, B and C regions are the outside, metadevices and object regions, respectively.

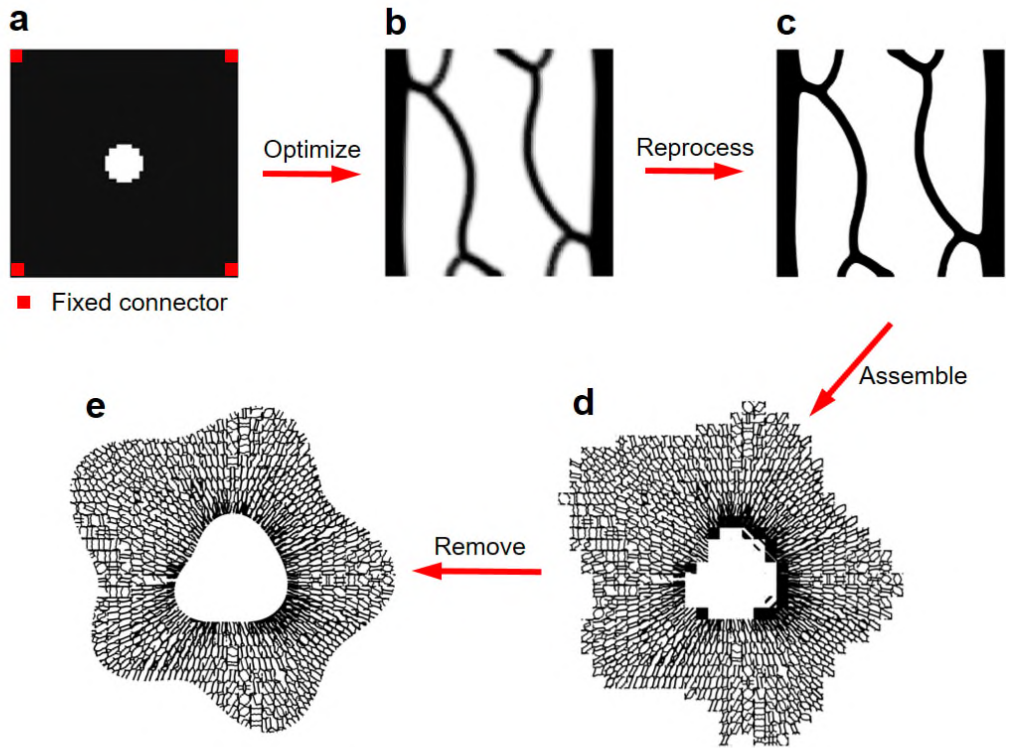

Supplementary Figure 9. Details in design and assembly of TFCs. (a) Initial structure of TFCs. **(b)** Optimized structure of a TFC. **(c)** Reprocessed structure of the optimized TFC. **(d)** The assembled structure of all the TFCs. **(e)** The final robustly printable freeform thermal metamaterial.
